# Genofunc: genome annotation and identification of genome features for automated pipelining analysis of virus whole genome sequences

**DOI:** 10.1186/s12859-023-05356-3

**Published:** 2023-05-30

**Authors:** Xiaoyu Yu

**Affiliations:** Institute of Evolutionary Biology, University of Edinburgh, Edinburgh, EH9 3FL Scotland, UK

**Keywords:** Genome annotation, Bioinformatics pipeline, Phylogenetics, Virus

## Abstract

**Background:**

Viral genomics and epidemiology have been increasingly important tools for analysing the spread of key pathogens affecting daily lives of individuals worldwide. With the rapidly expanding scale of pathogen genome sequencing efforts for epidemics and outbreaks efficient workflows in extracting genomic information are becoming increasingly important for answering key research questions.

**Results:**

Here we present Genofunc, a toolkit offering a range of command line orientated functions for processing of raw virus genome sequences into aligned and annotated data ready for analysis. The tool contains functions such as genome annotation, feature extraction etc. for processing of large genomic datasets both manual or as part of pipeline such as Snakemake or Nextflow ready for down-stream phylogenetic analysis. Originally designed for a large-scale HIV sequencing project, Genofunc has been benchmarked against annotated sequence gene coordinates from the Los Alamos HIV database as validation with downstream phylogenetic analysis result comparable to past literature as case study.

**Conclusion:**

Genofunc is implemented fully in Python and licensed under the MIT license. Source code and documentation is available at: https://github.com/xiaoyu518/genofunc.

**Supplementary Information:**

The online version contains supplementary material available at 10.1186/s12859-023-05356-3.

## Introduction

In the past decades, next generation sequencing and the evolution of computational biology have brought forth traction in the field of large scale genomics with public health implications [1, 2] alongside epidemiological surveillance and control [3, 4]. Viral phylodynamics uses genomic and epidemiological data in combination with mathematical modelling allowing estimation of phylogenies inferring valuable information of viruses through time and geographical locations based on metadata [5]. Currently, with the Covid-19 pandemic, the field of viral phylodynamics and automated bioinformatic pipelines have accelerated with a plethora of toolkits created to achieve this in real-time as a way of pandemic response [6–8].

Human immunodeficiency virus (HIV) is a rapidly evolving RNA virus being the largest viral pandemic pre-Covid with more than 37 million infected individuals in 2020 based on UNAIDS [9]. Initiatives such as PANGEA (Phylogenetics And Networks for Generalised Epidemics in Africa) have laid the groundwork in an attempt to both increase knowledge through large-scale sequencing to tackle HIV through prevention and treatment measures in key populations in an attempt to slow down the pandemic [10, 11]. Los Alamos HIV database also provides a platform with diversified whole genome HIV sequences from around the world with sample times over decades [12].

Here, we present Genofunc, a tool mainly for the annotating of raw sequences with protein coding features by aligning them in-frame based on reference sequence(s). In combination with several other command line functions such as feature extraction and filtering, key genomic features can be extracted for downstream phylogenetic analysis through tools such as Nextstrain augur [13] or BEAST [14].

Genofunc is an easily utilizable single command line tool fully scripted using python3 and can be installed through Python Package Index (PyPI) through pip install Genofunc. We test the Genofunc pipeline using whole genome HIV sequences downloaded from the Los Alamos HIV database as well as benchmark the estimated annotations from Genofunc against the Los Alamos annotated information. We validate the pol dataset extracted and infer the HIV root date and location with estimated clock rate using BEAST and compare results to past literature [15, 16].

## Implementation

**Fig. 1:**
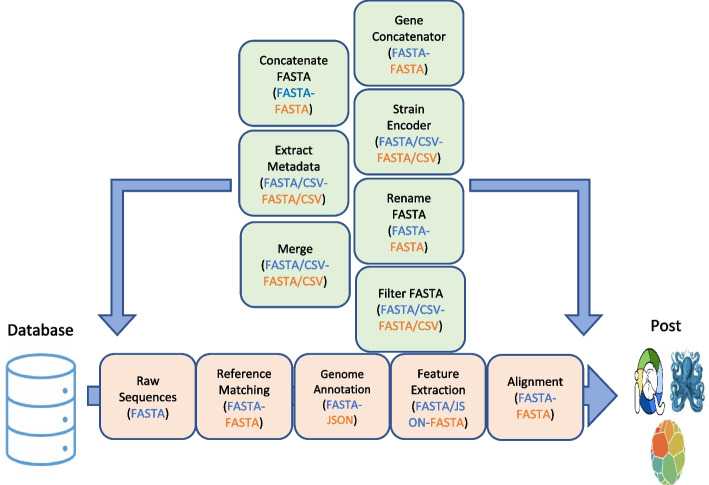
Function workflow in processing raw HIV sequences to aligned sequences through Genofunc scripts ready for phylogenetic analysis using tools such as augur or BEAST. Light orange boxes contain the main workflow for processing raw sequence file to phylogeneticly ready alignment with input/output file type at each step declared within the brackets split by a hyphen respectively. The main functions are, Reference_matcher matching raw HIV sequences to the closest annotated sequence within a pre-constructed reference file. Genome_annotator map the raw sequence to the best reference from the output from reference_matcher and annotate the mapped sequence with the information on the best reference. Feature_extractor allows users to extract gene regions based on the annotated information from genome_annotator. Sequence alignment can be done through group_align which aligns multiple groups of sequences against a consensus sequence and then concatenated together for more efficient alignment or use any other alignment tools such as MAFFT [19]. The aligned file can be feed to BEAST [14] or augur [13] for phylogenetic post-analysis. Other task orientated functions (green box) can be added to the main pipeline at any phase of the workflow if the input (blue) and output (orange) files fit the up and downstream functions

To allow easy utility and installation of Genofunc, the toolkit can be installed through GitHub or pip. This toolkit is fully written in python3.9 with functions constructed in a sequential fashion for easy pipelining mechanics of manipulating large raw HIV whole genome sequence datasets. Genofunc includes single command line functions consisting of two main components, FASTA/metadata file manipulation and HIV sequence processing (Fig. 1). For the prior component, single-task functions are scripted for easy manipulation of FASTA files and metadata files in CSV or TSV formats. The general functions include FASTA concatenation and filtering, metadata extraction and merging, sequence ID encoding and renaming etc. The latter functions are specific for HIV sequences including matching raw sequence to an existing reference file created by the user using minimap2 (reference_matcher) [17]; annotating raw sequence to the closest reference genome through local pairwise alignment using parasail (genome_annotator) [18] and finally extracting gene regions of interest based on annotated information (feature_extractor). Documentation and example command line references can be found on the Genofunc GitHub page.

## Results

To identify the versatility and accuracy of the annotation and extraction of gene features using Genofunc, we downloaded and tested on three viral datasets, the West Nile virus, the Zaire ebolavirus and the Monkeypox virus, from GenBank and one HIV-1 dataset from Los Alamos. All complete sequences with fully annotated gene features were used as the test dataset summing up to 1211, 512, 914 and 6030 sequences respectively. Accession ID HQ596519, KJ660348 and NC_003310 were used as reference sequences for West Nile virus, Zaire ebolavirus and Monkeypox virus according to past literature [19–21] while a reference list of 237 sequences was selected consisting of the newest sequence from each subtype for HIV-1. The pipeline shown in Fig. 1 was performed without the final alignment step with all gene features extracted for all four datasets. Due to the large number of ORFs contained within the Monkeypox virus which many does not have well defined functions, we selected the first 6 genes which are associated with virulence and immune evasion within literature as a subset to represent the feasibility of Genofunc feature identification function for larger DNA genomes [22]. Similarly, complete genome sequences were imported, annotated based on the same reference and extracted using Geneious Prime using default settings [23]. Local alignments were performed for each viral sequence gene feature extracted by Genofunc and Geneious Prime to calculate a match ratio (sum of matching nucleotides/alignment length) against the corresponding annotated gene regions on GenBank. A summary comparing the performance of Genofunc against Geneious for the four viral datasets in terms of accuracy and run time are shown in Table 1.

**Table 1:** Accuracy and run time comparison between Genofunc and Geneious for feature identification and extraction

Software	Virus	Number of sequences	Accuracy*	Run time**
Genofunc	Zaire ebolavirus	512	0.9993	± 19 min
Geneious			0.9996	± 4 min
Genofunc	West Nile virus	1211	0.9827	± 11 min
Geneious			0.9827	± 10 min
Genofunc	HIV-1	6030	0.9969	± 31 min
Geneious			0.9905	± 2 h 44 min
Genofunc	Monkeypox virus	914	0.9800	± 15 h 23 min
Geneious			0.9999	± 52 min

*Accuracy here represents the average match ratio of all gene feature extracted for the virus annotated. Match ratio is calculated as the number of matching nucleotides over alignment length between estimated gene feature based on software against GenBank/Los Alamos annotation

**Both Geneious and Genofunc were benchmarked using MacBook Pro 2.6 GHz 6-core i7 32 GB DDR4 memory laptop

Next, we test the efficiency of the Genofunc pipeline using 16,832 whole genome sequences from the Los Alamos HIV database [12]. Through filtering of missing metadata and removing duplicated sequences, we retained a raw dataset of 10,358 sequences ranging over a variety of subtypes and countries in an attempt to estimate the root of HIV-1. Following a similar pipeline to the benchmarking test, the pol gene region were extracted from the raw Los Alamos HIV-1 dataset using Snakemake [24] combining the Genofunc functions reference_matcher, genome_annotator, feature_extractor and filter_fasta resulting in a dataset of 10,293 pol sequence. 65 sequences were filtered out with coverage of less than 95% un-ambiguous sites (non-N) and/or length span of under 2500 nucleotide bases. Alignment was done using MAFFT [25] under default settings and regions with over 50% gaps were masked to create the final aligned dataset.

Phylogenetic analysis was done in another pipeline using augur [13] starting with a phylogenetic tree inferred using IQTREE with substitution model GTR + R6 [26, 27]. Timed phylogeny was inferred using TREETIME [28] with a clock rate of 0.001075 pre-estimated using BEAST on the 239 reference sequences [14]. A clock filter of 4.5 inter-quartile range was set to remove any sequence outliers which does not fit the clock regression model. Ancestral traits reconstruction was done using TREETIME for the estimation of root location. The inferred root date for HIV-1 is 1905 based on 9868 pol sequences resulting from sequences being removed as outliers not fitting to the molecular clock given. The ancestral location inferred is Africa between Cameroon and the Democratic Republic of Congo reflecting a similar region in past literature [15, 16] (Additional file 1: Fig. S2).

## Discussion

Although Genofunc was written originally written for processing HIV genome sequences, the package can be easily utilised for other viral sequences with a given reference genome. This is shown through the benchmarking test of different viral datasets against another annotation tool Geneious and comparable to the annotation information on GenBank/Los Alamos. The results are robust not only for a diverse viral dataset such as HIV-1 using a short list of reference sequences but also proving true for viruses that are annotated using only a single reference for estimating gene feature coordinates with high accuracy over all genes analysed. Genofunc also remains robust for annotating larger DNA genomes but at a slightly lower accuracy compared to shorter RNA viruses for extracting specific gene features (Additional file 1: Fig. S1). However, it is worth taking note that the reference(s) chosen should be well annotated and represent the input raw dataset for better accuracy on estimated gene features.

We believe that based on our case study, Genofunc could be easily used on all viruses for the annotation and identification of gene features especially suitable for processing large raw sequence datasets. The setup of the pipeline using Genofunc in this case study also showed efficiency and simplicity in annotating and extracting key genomic regions from raw sequences which can be used in all forms of applications. This is shown through the runtime comparison to Geneious whereby Genofunc scale better with increase in dataset size but poorly for long viral genome sequences. Therefore, the current version of Genofunc is highly recommended for large scale short genome analysis (< 50 Kb). Future development of Genofunc lies in improving algorithm for reducing runtime in analysing longer genomes, improving accuracy in annotating gene features and correcting artefactual frameshifts that may be due to errors in sequencing or consensus genome calling pipelines.

In summary, Genofunc is a single command line toolkit suitable for constructing an automated pipeline to process large raw virus sequence datasets efficiently and readily for large scaled phylogenetics. Genofunc is open source and available with documentation at https://github.com/xiaoyu518/genofunc.

## Supplementary Information

**Additional file 1.** Supplementary Figures.

## Data Availability

All data used in this research can be found on the Los Alamos HIV database found at https://www.hiv.lanl.gov/content/index and GenBank on https://www.ncbi.nlm.nih.gov/. Project name: Genofunc. Project home page: https://github.com/xiaoyu518/genofunc. Operating system(s): iOS/Linux. Programming language: Python. Other requirements: None. License: MIT. Any restrictions to use by non-academics: None.
