# Supplementary material for: Genofunc: genome annotation and identification of genome features for automated pipelining analysis of virus whole genome sequences
Source: BMC Bioinformatics. 2023 May 30;24:218. doi: 10.1186/s12859-023-05356-3 (PMC10227794; doi:10.1186/s12859-023-05356-3)
Supplement: Supplementary file 1 — Additional file 1. Supplementary Figures. [file 12859_2023_5356_MOESM1_ESM.docx]

**Genofunc: Genome annotation and identification of genome features for automated pipelining analysis of raw virus whole genome sequences.**

**Supplementary Figures**


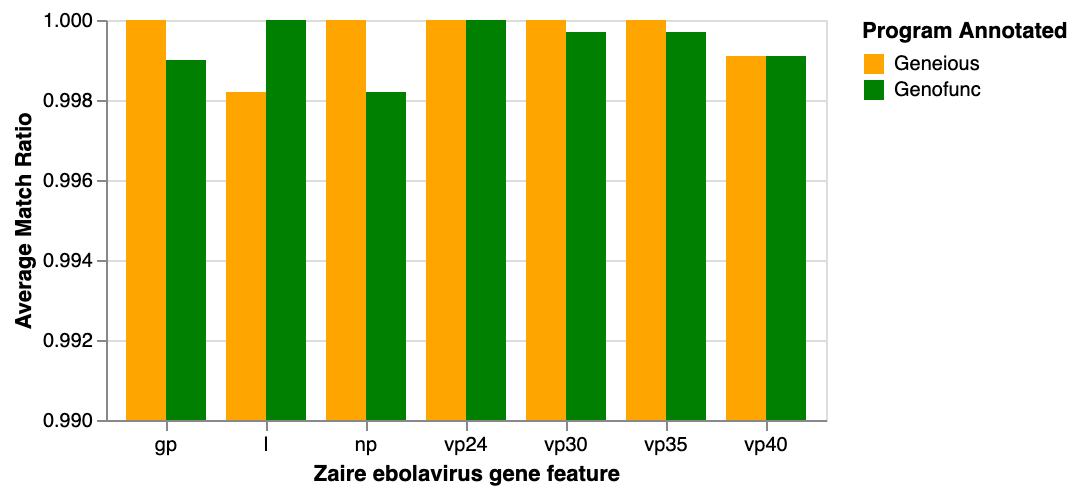

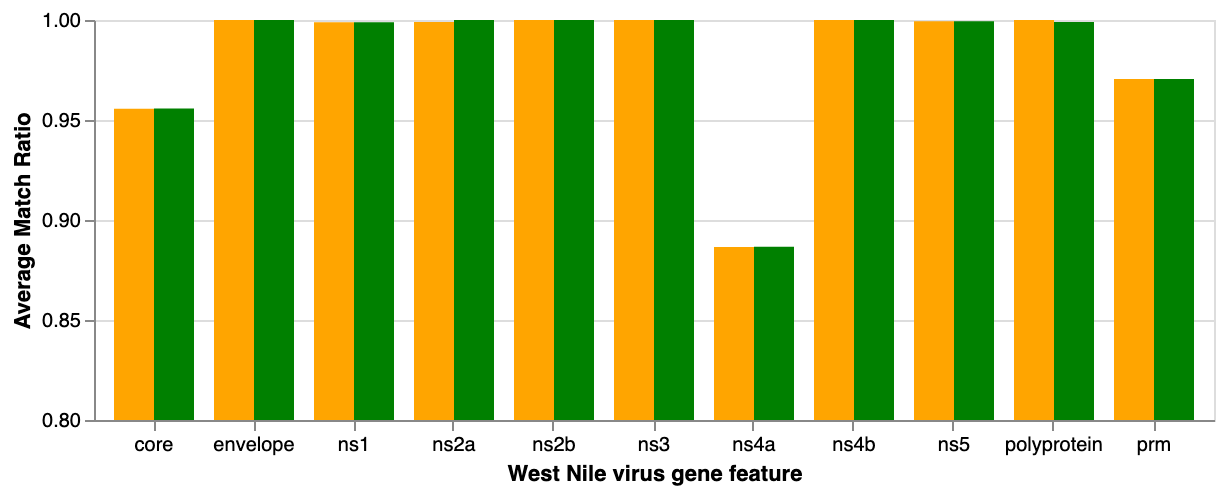

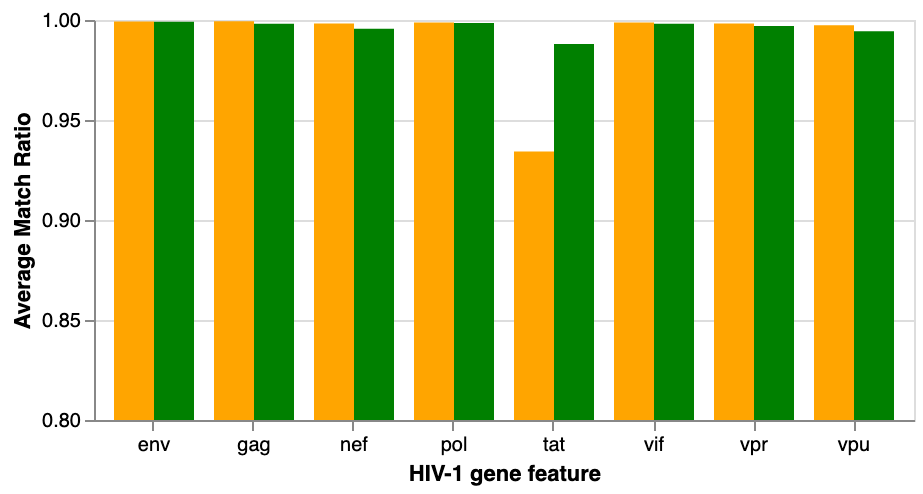

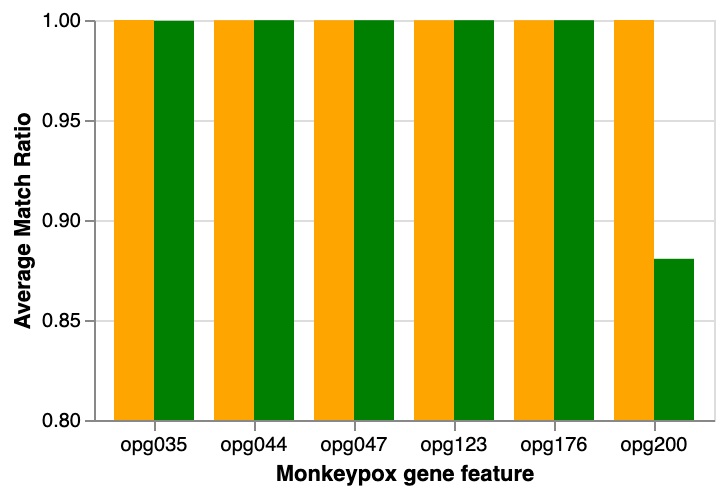


Supplementary Figure 1. Gene features extracted using Genofunc and Geneious are compared to corresponding Genbank annotated gene regions based on match ratio. Match ratios are calculated as matching nucleotide bases over alignment length. The bar chart displays the average match ratio for all Genofunc estimated gene features for each viral dataset on par with Geneious Prime with high accuracy compared to the annotated regions on GenBank/Los Alamos. Overall Genofunc performed on par with Geneious with high accuracy in annotating gene features across all viruses (poorest performance is Monkeypox virus with gene feature opg200 at 88%). Both Genofunc and Geneious fall short for gene features such as ns4a, prm and core, however, this is not due to poor estimates of the region but the matching annotation on GenBank having additional regions to the feature. For example, a few West Nile virus complete sequences in GenBank include ns4a with a 2k protein feature making ns4a alone shorter than the Genofunc and Geneious estimated region assuming both features are one. However, such problem can be prevented by using additional references with such information.


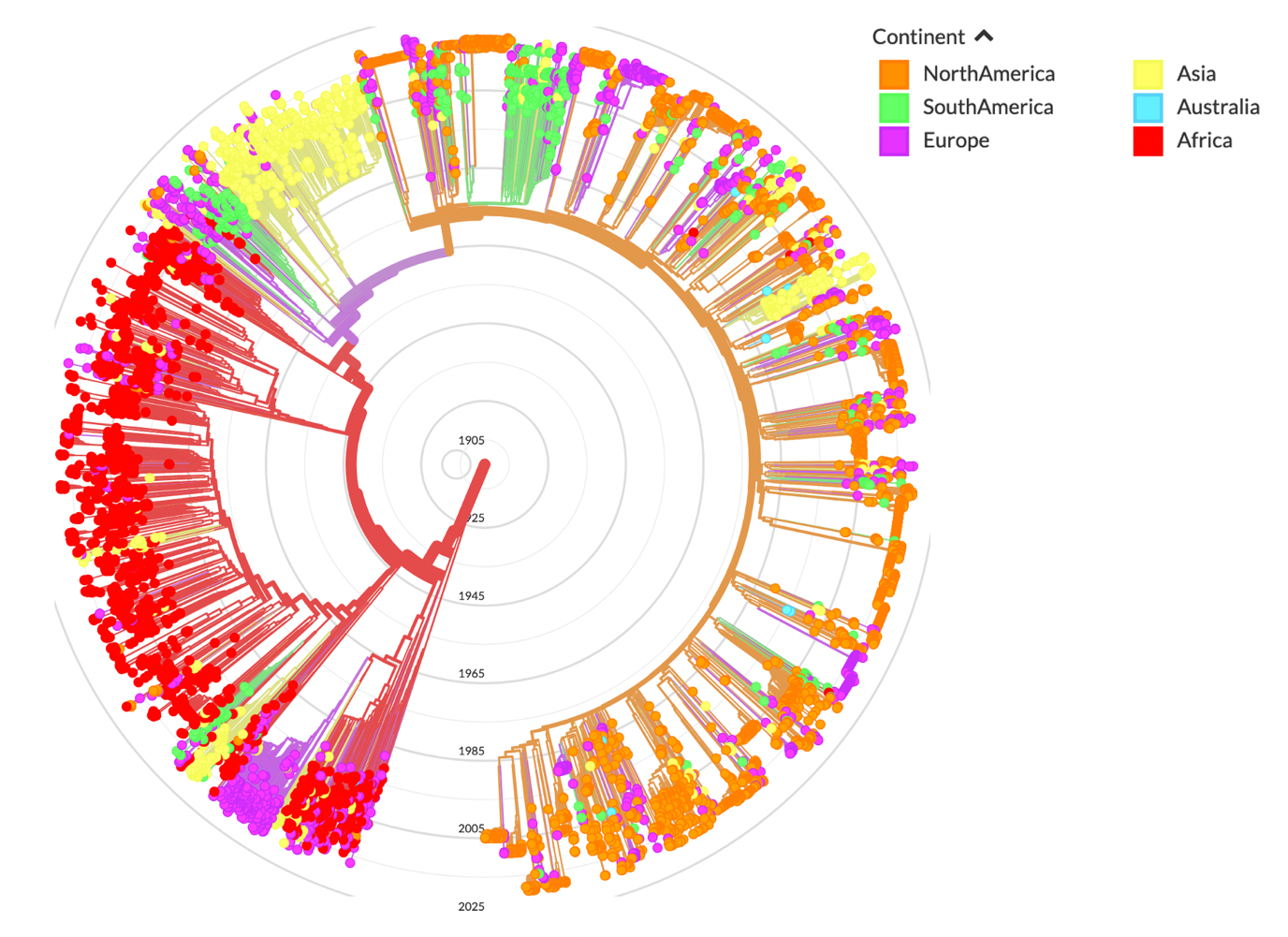


Supplementary Figure 2. Inferred phylogenetic tree using the HIV-1 pol Los Alamos dataset projected using auspice. The root date is estimated at 1905 with the likely source location in the Democratic Republic of Congo (DRC). Tree colour coded by continent as shown by the top left corner.
